# Nutritional Intake and the Risk for Non-Alcoholic Fatty Liver Disease (NAFLD)

**DOI:** 10.3390/nu11030588

**Published:** 2019-03-11

**Authors:** Jörn M. Schattenberg, Ina Bergheim

**Affiliations:** 1 Department of Internal Medicine I, University Medical Centre of the Johannes Gutenberg-University Mainz, 55131 Mainz, Germany; 2 RA Molecular Nutritional Science, Department of Nutritional Sciences, University of Vienna, Althanstr.14, 1090 Vienna, Austria; ina.bergheim@univie.ac.at

The prevalence of non-alcoholic fatty liver disease (NAFLD) is rising worldwide, and it is estimated that approximately one billion individuals may be afflicted with NAFLD globally [1,2]. This epidemic has created a large demand for therapeutic interventions in order to treat patients at risk for the development of end-stage liver disease. As NAFLD is a slowly progressive disease along a spectrum that covers (i) hepatic steatosis defined as non-alcoholic fatty liver (NAFL), (ii) inflammatory non-alcoholic steatohepatitis (NASH) and can lead to (iii) end-stage liver disease including cirrhosis and hepatocellular carcinoma, there seems to be a large window of opportunity to counsel and provide care to these patients. Albeit the relatively slow progression of the liver phenotype in most patients—ranging from 7 to 14 years per stage of hepatic fibrosis—cardiovascular-mortality remains the leading cause of mortality in NAFLD patients [3]. This feeds into the hypothesis that NAFLD is a multisystem, metabolically-driven, inflammatory disease affecting several target organs including, but crucially not limited to, the liver. Accumulating evidence supports a role of adipose tissue inflammation, changes in the gut microbiota and intestinal barrier function, that act directly—e.g., through short-chain fatty acids, increased translocation of bacterial endotoxins or lipotoxins—or indirectly—e.g., through the regulation of insulin sensitivity and resulting hyperlipidemia—on the liver. While there is ample evidence that lifestyle interventions are effective in patients with NASH, the extent and the composition of the diet are less defined, and many patients fail to adhere to complex changes of daily life, thus creating a high unmet need to provide simple nutritional regimens that address the above-named pathomechanisms. 

The role of specific macronutrients, such as saturated fatty acids or sugars e.g., sucrose and fructose, and also certain dietary patterns, like the so-called ‘Western-type dietary patterns’ composed of a combination of highly processed foods, sweets, candy, and sugar-sweetened beverages as well as red meat, refined grains, and high dairy fat, has been deemed critical to the onset and progression of the disease [4,5] (see Figure 1). Indeed, in overweight children with early stages of the disease, not only a general overnutrition but rather an overnutrition with a diet rich in sugar mainly derived from sweetend beverages seems to be critical in the development of NAFLD [6]. This was associated with increased bacterial endotoxin levels further suggesting that even in very early stages of the disease intestinal barrier function may be already impaired. Furthermore, results of this study also suggest that even a very moderate dietary intervention focusing soley on a reduction of overall fructose intake in children may have beneficial effects on bacterial endotoxin levels in healthy young adults [6]. Supporting the hypothesis that altering the dietary pattern in NAFLD patients may impact disease development through changes in that intestinal barrier function was also shown in a study that counseled to increase the intake of whole-wheat bread, pasta, and cereal as well as brown rice alongside an increase in vegetable and fruit intake [7]. Indeed, in this study improvement of alanine aminotransferase (ALT) and aspartate aminotransferase (AST) in serum was associated with lower markers of intestinal permeability in serum even when the BMI was not altered.

Regarding the fat intake and especially fatty acid composition of a diet, the findings are controversial. Lipidomic studies suggest that lipid profile and herein especially phospholipid composition of patients with early and more progressed stages of NAFLD are markedly altered when compared to healthy controls and that this may be related to the genetic background of patients [8,9]. However, the results of animal studies suggest that hepatic lipid export may also be critical [10]. In a placebo controlled dietary intervention study focusing on improving omega-3 index and omega-6 to omega-3 fatty acid ratio through supplementing a high concentrate omega-3 fatty acid preparation (MF4637) beneficial effects found on liver fat content and transaminase activity of NAFLD patients were similar to those of the placebo group treated with olive oil [11].Still, omega-3 index and absolute values of red blood cell eicosapentaenoic acid and docosahexaenoic acid were only significantly altered in those patients receiving the omega-3 fatty acid preparation. These data further suggest that altering only omega-3 fatty acid concentration by itself may not be sufficient to improve the liver status, superior to what can be achieved by altering fat intake towards an increased intake of vegetable oils. Also, considering the cardiovascular risk of patients with NAFLD, it is noteworthy that a recent trial on supplementation with marine n-3 fatty acids was not effective in reducing the number of major cardiovascular events [12]. Results of mouse studies further support the hypothesis that the source of fat and the content of unsaturated fatty acids in diet may be critical in the development of NAFLD. For instance, it was shown that a high fat diet rich in soybean oil -derived omega-6 fatty acids may trigger disease progression [13] and thus further underlines the necessity to carefully consider recommendations provided to patients with NAFLD regarding fat intake. 

Not only the intake and circulating levels of macronutrients are deemed to be critical to the development of NAFLD. Indeed, it has been suggested that abnormal serum levels of vitamins are also frequently encountered in patients even with early stages of the disease. For instance, it has been shown that low levels of folate and vitamin B12 are significantly related to severity of NASH [14]. However, the current study did not examine if this related to changes in the dietary pattern with low intake, malabsorption or an increased metabolism of these vitamins, and no implications for disease progression, or even therapeutic application can be made.

Taken together and as presented in the accompanying special issue on nutrition in NAFLD, dietary patterns markedly impact the development and progression of NAFLD and are worthwhile to be addressed in therapeutic interventions. Changes of dietary pattern taking dietary habits of patients into account rather than reducing the overall caloric intake and increasing physical activity may indeed be an alternative or a favorable therapeutic and preventive approach for patients with NAFLD, as adherence is critical for long-term success.

## Figures and Tables

**Figure 1:**
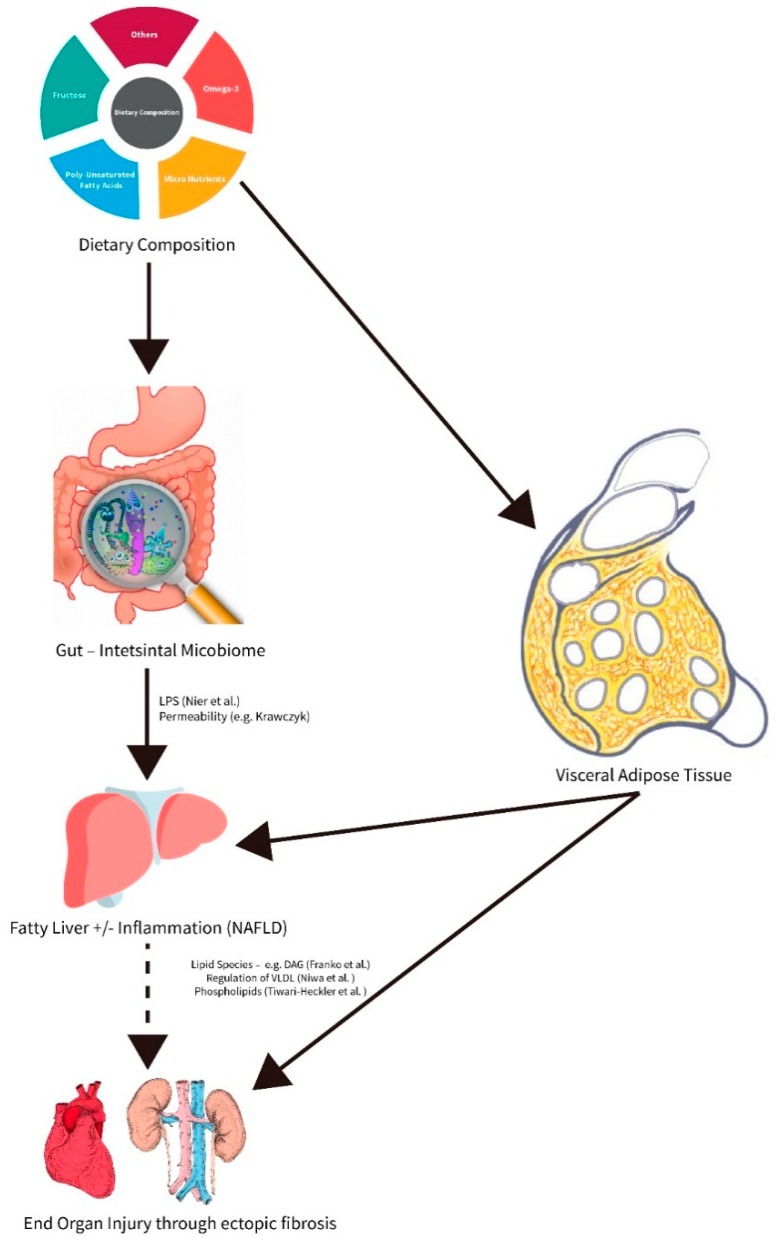
Role of macro- and micronutrients in affecting hepatic and extrahepatic inflammation and fibrosis during the progression of non-alcoholic fatty liver disease (NAFLD).
